# Biofilm spatial organization by the emerging pathogen *Campylobacter jejuni*: comparison between NCTC 11168 and 81-176 strains under microaerobic and oxygen-enriched conditions

**DOI:** 10.3389/fmicb.2015.00709

**Published:** 2015-07-13

**Authors:** Hana Turonova, Romain Briandet, Ramila Rodrigues, Mathieu Hernould, Nabil Hayek, Alain Stintzi, Jarmila Pazlarova, Odile Tresse

**Affiliations:** ^1^SECALIM UMR1014, Institut National de la Recherche AgronomiqueNantes, France; ^2^LUNAM Université, Oniris, Université de NantesNantes, France; ^3^Department of Biochemistry and Microbiology, Faculty of Food and Biochemical Technology, University of Chemistry and TechnologyPrague, Czech Republic; ^4^MICALIS UMR1319, Institut National de la Recherche AgronomiqueMassy, France; ^5^Department of Biochemistry, Microbiology and Immunology, Faculty of Medicine, University of OttawaOttawa, ON, Canada

**Keywords:** *Campylobacter jejuni*, biofilm, CLSM, oxidative stress, CosR

## Abstract

During the last years, *Campylobacter* has emerged as the leading cause of bacterial foodborne infections in developed countries. Described as an obligate microaerophile, *Campylobacter* has puzzled scientists by surviving a wide range of environmental oxidative stresses on foods farm to retail, and thereafter intestinal transit and oxidative damage from macrophages to cause human infection. In this study, confocal laser scanning microscopy (CLSM) was used to explore the biofilm development of two well-described *Campylobacter jejuni* strains (NCTC 11168 and 81-176) prior to or during cultivation under oxygen-enriched conditions. Quantitative and qualitative appraisal indicated that *C. jejuni* formed finger-like biofilm structures with an open ultrastructure for 81-176 and a multilayer-like structure for NCTC 11168 under microaerobic conditions (MAC). The presence of motile cells within the biofilm confirmed the maturation of the *C. jejuni* 81-176 biofilm. Acclimation of cells to oxygen-enriched conditions led to significant enhancement of biofilm formation during the early stages of the process. Exposure to these conditions during biofilm cultivation induced an even greater biofilm development for both strains, indicating that oxygen demand for biofilm formation is higher than for planktonic growth counterparts. Overexpression of cosR in the poorer biofilm-forming strain, NCTC 11168, enhanced biofilm development dramatically by promoting an open ultrastructure similar to that observed for 81-176. Consequently, the regulator CosR is likely to be a key protein in the maturation of *C. jejuni* biofilm, although it is not linked to oxygen stimulation. These unexpected data advocate challenging studies by reconsidering the paradigm of fastidious requirements for *C. jejuni* growth when various subpopulations (from quiescent to motile cells) coexist in biofilms. These findings constitute a clear example of a survival strategy used by this emerging human pathogen.

## Introduction

*Campylobacter* has emerged as the leading cause of bacterial foodborne infections in developed countries (Epps et al., 2013; Golz et al., 2014). The resulting disease in humans, campylobacteriosis, is characterized by acute enteritis with the presence of blood and leukocytes in a stool, abdominal pain, and fever (Cameron et al., 2012; Lu et al., 2012). It is also associated with late onset complications such as Guillain-Barré syndrome, its variant Miller-Fisher syndrome (Salloway et al., 1996; Nachamkin et al., 1998; Kudirkiene et al., 2012), and inflammatory bowel diseases (Kaakoush et al., 2014). The underlying molecular mechanisms responsible for its pathogenesis, persistence, and survival seem to be unique to *Campylobacter* as compared to other invasive foodborne bacterial pathogens (*Listeria monocytogenes, Salmonella enterica*, and *Staphylococcus aureus*). These features might result from high level of genomic polymorphism, restricted catabolic capacity, self-regulation and deregulation of genes, and other undefined survival routes.

The main reservoir of *Campylobacter* is the intestinal tract of birds and other endothermic animals, especially livestock. It is primarily isolated from poultry and, to a lesser extent, pork and beef. The infection of the human host is generally caused by the consumption of undercooked and mishandled poultry or by cross-contamination of cooking tools and fresh vegetables (Butzler, 2004; Guyard-Nicodeme et al., 2013). A significant increase in the prevalence of campylobacteriosis cases has been observed over the past 5 years in the EU, based on quantitative epidemiological analyses from farms to retail outlets (EFSA, 2012, 2013). A baseline survey, conducted in 28 European countries in 2010, indicated that 71.2% of broiler batches and 75.8% of broiler carcasses were contaminated by *Campylobacter* (EFSA, 2010). These data were reinforced by an in-depth analysis over a 3-year period at the UK-wide level showing that in over 37 abattoirs (representing almost 90% of the total UK slaughter throughput), 79.2% of the slaughter batches were positive for *Campylobacter* (Lawes et al., 2012). In addition, 87.3% of the broiler carcasses were contaminated by *Campylobacter* with 27.3% of them showing a load over 1000 cfu.g^−1^ (Powell et al., 2012). In the USA, 168 pathogen-food combinations of 14 major pathogens across 12 food categories were compared (Batz et al., 2012). The combination “*Campylobacter*-poultry” reached the first rank in terms of annual disease burden including illnesses, hospitalizations, deaths, and costs. Overall, these exhaustive data on *Campylobacter* contamination indicate that this microorganism can survive outside of its reservoir through breeding farms, slaughterhouses and food processing, defying environmental conditions, and human defense mechanisms.

The main pathogenic species, *Campylobacter jejuni*, has been isolated in more than 80% of the campylobacteriosis cases (Moore et al., 2005). Being an obligate microaerophilic bacterium, *Campylobacter* has to develop adaptation strategies to survive oxidative conditions from food environments and macrophage attacks. It has been suggested that adhesion to surfaces and formation of biofilms could be one of the strategies used to maintain *C. jejuni* survival (Nguyen et al., 2012). Moreover, the bacterium can be sheltered in mixed species biofilms (Sanders et al., 2007; Ica et al., 2011). *C. jejuni* can form three different types of biofilm: (i) a structure attached to an abiotic surface, (ii) aggregates floating in the liquid culture, or (iii) a pellicle formed at the gas/liquid interface (Joshua et al., 2006). Biofilm formation occurs within 48 h of cultivation with cell detachment becoming prominent after a prolonged cultivation time (Sanders et al., 2007; Ica et al., 2011). In line with other biofilm-producing foodborne bacteria, the substratum composition and its physicochemical properties influence the biofilm formation of *C. jejuni* (Nguyen et al., 2011). These properties could play an important role in the early stages of biofilm formation when cells adhere to the surface. This assumption is supported by a variation in adhesion rates to inert surfaces, such as nitrocellulose membrane, glass, and stainless steel (Joshua et al., 2006; Kalmokoff et al., 2006; Gunther and Chen, 2009). The wide range of adhesion capability in *Campylobacter* spp. also raises the question of biological fitness among strains, in regards to their ability to attach irreversibly to a surface and initiate biofilm formation (Joshua et al., 2006; Sulaeman et al., 2010; Teh et al., 2010).

Molecular mechanisms regulating biofilm formation of *C. jejuni* are still poorly understood. So far, genes described to be involved in the process include those responsible for cell motility (*flaA, flaB, flaC, flaG, fliA, fliS*, and *flhA*; Joshua et al., 2006; Kalmokoff et al., 2006; Reeser et al., 2007; Reuter et al., 2010), cell surface modifications (*peb4, pgp1*, and *waaF*; Asakura et al., 2007; Naito et al., 2010; Frirdich et al., 2014), quorum sensing (*luxS*; Reeser et al., 2007), and stress response (*ppk1, spoT, cj1556, csrA, cosR*, and *cprS*; Candon et al., 2007; Fields and Thompson, 2008; McLennan et al., 2008; Svensson et al., 2009; Gundogdu et al., 2011; Oh and Jeon, 2014). It was found that biofilm formation is flagellum-mediated as the first step of the process—cellular adhesion—requires presence of flagella, although its functionality is not crucial for the biofilm initiation (Svensson et al., 2014). Other components essential for development of biofilm structure are extracellular DNA (eDNA) and DNA-binding protein Dps, whose presence is required for proper formation of microcolonies and structuralization of biofilm (Svensson et al., 2014; Brown et al., 2015). Genes regulating biofilm formation were not fully identified so far. Experiments using knock-out and knock-down mutants of various regulators revealed several genes influencing the process of biofilm formation. Except of aforementioned motility apparatus regulated by *flhA* (Kalmokoff et al., 2006), and functional quorum sensing *luxS* (Reeser et al., 2007), other regulators involved mostly in stress response were found to be critical for biofilm formation. Interestingly, while mutants lacking genes responsible for oxidative stress response such as *cj1556* and *csrA* were defective in biofilm formation (Fields and Thompson, 2008; Gundogdu et al., 2011), knock-out/down of genes responsible for general stress response (*spoT, ppk1*, and *cprS*) resulted in increased biofilm formation suggesting that the process represents alternative pathway of stress defense in *Campylobacter* (Candon et al., 2007; McLennan et al., 2008; Svensson et al., 2009). Another regulator possibly involved in biofilm formation is gene *cosR*. This orphan two-component system (TCS) was recently discovered to be involved in the regulation pathway of ROS detoxification in *C. jejuni* (Hwang et al., 2011, 2012). It was previously reported that CosR regulates transcription of 93 different genes in *C. jejuni* (Hwang et al., 2012), it is overexpressed in sessile cells (Kalmokoff et al., 2006) and was already shown to influence biofilm formation by regulation of alkyl hydroperoxide reductase *ahpC* (Oh and Jeon, 2014). All these facts suggest that CosR might play significant role in biofilm formation of *C. jejuni*.

So far, analyses of pure cultures have mostly been carried out in an optimal growth atmosphere and were focused on the strain NCTC 11168 (Kalmokoff et al., 2006; Ica et al., 2011). Using colorimetric assessment methods (Crystal violet and Congo red assays) for biofilm detection in glass tubes, Reuter et al. (2010) showed that aerobic cultivation enhanced *C. jejuni* NCTC 11168 biofilm. In a previous study, we have shown that the strain 81-176, grown under controlled oxygen-enriched conditions (19% O_2_, 10% CO_2_, and 71% N_2_), is able to overexpress membrane proteins involved in biofilm initiation and virulence process (Sulaeman et al., 2012). In this study, we compared the biofilm development of two *C. jejuni* strains responsible for human outbreaks (NCTC 11168 and 81-176), and the effect of dioxygen (O_2_) on biofilm development. The usage of controlled atmosphere eliminated other factors possibly affecting biofilm formation. It was therefore possible to explore whether the increase of biofilm formation in aerobic conditions could be attributed solely to the level of oxygen and if the trend of enhanced biofilm formation is present in other strain of *C. jejuni*. This was evaluated, for the first time, using specific biofilm parameters (maximum height, biomass volume, and ultrastructure) from confocal laser scanning microscopy (CLSM) analyses. This non-invasive sensitive technique has been used previously to examine *Campylobacter* cell morphology and viability (Chantarapanont et al., 2003; Lee et al., 2004; Jang et al., 2007; Ica et al., 2011) and bacterial interactions with live tissues (Mooney et al., 2003). The CLSM has also been used for the detection of *C. jejuni* in mixed species biofilms (Sanders et al., 2007; Ica et al., 2011). In the present work, the impact of pretreatment and cultivation of cells in oxygen-enriched conditions (OEC) on *C. jejuni* biofilm formation and its ultrastructural organization was investigated in comparison with cells cultivated in microaerobic conditions (MAC). In addition, analyses using an overexpressing *cosR* transformant were performed to determine the role of this regulator in *C. jejuni* biofilm development.

## Results

### Biofilm development and architecture

**Figure 1:**
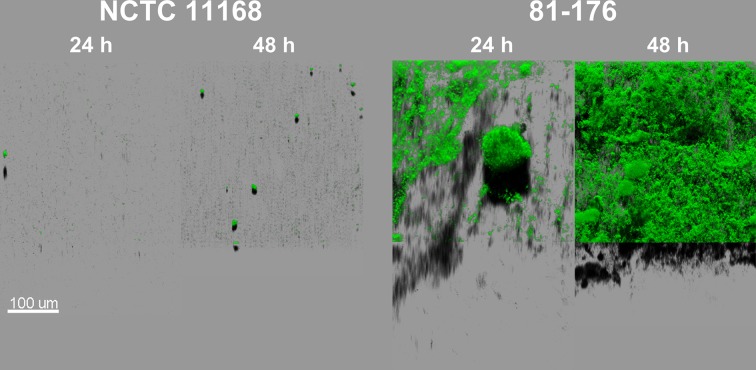
***C. jejuni*** **NCTC 11168 and 81-176 biofilm architecture and development are different after incubation for 24 and 48 h in MAC (5% O_2_, 10% CO_2_, 85% N_2_)**. The CLSM images represent an aerial view of biofilm structures with the shadow projection at the bottom. The structures were visualized using Syto 9, an intercalating agent staining the nucleic acids.

Two *C. jejuni* strains, NCTC 11168 and 81-176, were chosen in order to explore their biofilm formation capacities using CLSM with Syto 9 staining. This cell-permeable dye emits fluorescence after binding to nucleic acids and therefore allows the visualization of cells and any extracellular DNA present in the biofilm matrix. Both strains were able to form biofilm within 24 h of cultivation (Figure 1). At the initial stages of biofilm formation, cells gathered in clusters partially attached to the surface, forming finger-like structures. After 48 h, most of the biofilm mass remained attached to the bottom of the well. The biofilm structure evolved during the time of cultivation, increasing in both maximum height and biomass volume for both strains. However, 81-176 formed more biofilm than the NCTC 11168 strain (after 48 h: 233.33 ± 64.63 and 130.67 ± 14.70 μm, respectively, for the maximum height; 42.3 × 10^5^ ± 5.7 × 10^5^ and 0.4 × 10^5^ ± 0.09 × 10^5^ μm^3^, respectively, for the biomass volume; *n* = 3). In addition, unlike NCTC 11168, the biofilm of the 81-176 strain exhibited a pronounced open ultrastructure full of voids and channels, even after 96 h of incubation (data not shown). As growth rates of both strains were similar (μ_max_ = 0.69 h^−1^ for NCTC 11168 and μ_max_ = 0.67 h^−1^ for 81-176), these differences in biofilm formation cannot be explained by different growth abilities. During the experiment, no formation of pellicle or floating aggregates was observed probably due to the cultivation in static conditions.

### Cell motility in biofilm

Motile cells, tracked using CLSM, were observed around or inside the biofilm structure after 48 h of cultivation (Supplementary Videos). However, the motility of cells differed according to their position in the biofilm structure. The highest number of motile cells was detected at the bottom of the well (Supplementary Videos 1, 3) moving more or less freely through the structure, while the motility and the number of motile cells decreased in the middle part of the biofilm (Supplementary Videos 2, 4). Furthermore, high number of motile cells was detected within the biofilm structure of 81-176 (Supplementary Videos 1, 2), whereas for NCTC 11168 the motile cells were detected mostly outside the biofilm (Supplementary Videos 3, 4).

### Effect of oxygen on biofilm formation of NCTC 11168 and 81-176 *C. jejuni* strains

**Figure 2:**
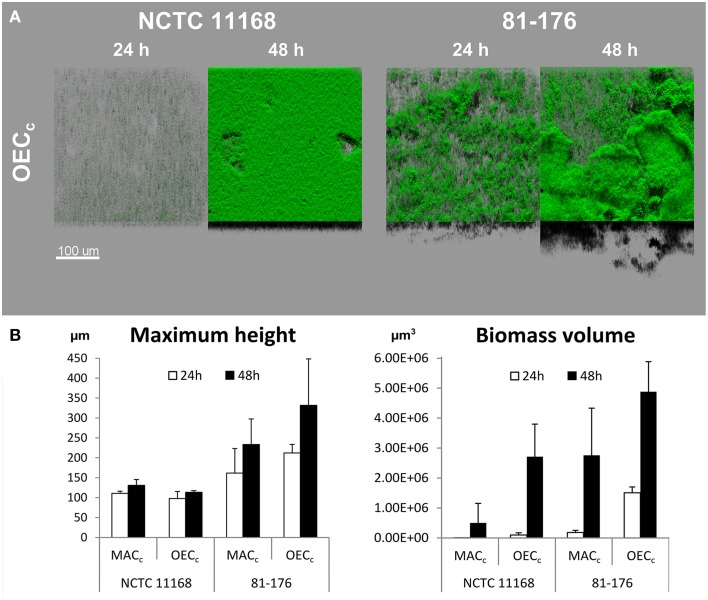
**Oxygen enhances biofilm development of** ***C. jejuni*** **NCTC 11168 and 81-176 after incubation for 24 and 48 h. (A)** The CLSM images represent an aerial view of the biofilm structures in OEC (19% O_2_, 10% CO_2_, 71% N_2_) with the shadow projection at the bottom. **(B)** The effect of cultivation time (24 h white bars, 48 h black bars) and OEC on biofilm formation of the two *C. jejuni* strains as expressed by maximum height and biomass volume. Results show the means and standard deviations of three replicates. Statistical data are presented in Supplementary Table 1.

Two different approaches were used to evaluate the effect of subinhibitory oxygen concentration on biofilm formation of two strains with different biofilm forming ability (NCTC 11168 and 81-176). Firstly, biofilms were cultivated under controlled oxygen-enriched conditions (OEC_*c*_) as described previously by Sulaeman et al. (2012). In OEC, the same concentration of CO_2_ (10%) as in MAC was maintained, while the O_2_ concentration was increased to a sublethal level (19% O_2_ in OEC vs. 5% in MAC). This enabled the evaluation of the effect of increased O_2_ concentration on biofilm development of *C. jejuni* regardless of its capnophilic nature requiring increased concentration of CO_2_. Biofilm volume of both strains was significantly increased (*P* < 0.01) when cultivated in OEC_*c*_ (Figure 2 and Supplementary Table 1). Incubation time and O_2_ concentration had a significant effect (*P* < 0.01) on increased biomass production in OEC_*c*_ when compared to MAC_*c*_. Interestingly, some significant differences in both maximum height and biomass volume (*P* < 0.01) remained between the two strains even after cultivation in OEC, with a greater biofilm development for 81-176 than for NCTC 11168, indicating that strain biology impacts biofilm formation (Supplementary Table 1). This was confirmed by formation of a denser compact biomass for NCTC 11168 biofilm while 81-176 induced more voids and open water channels across the biofilm.

**Figure 3:**
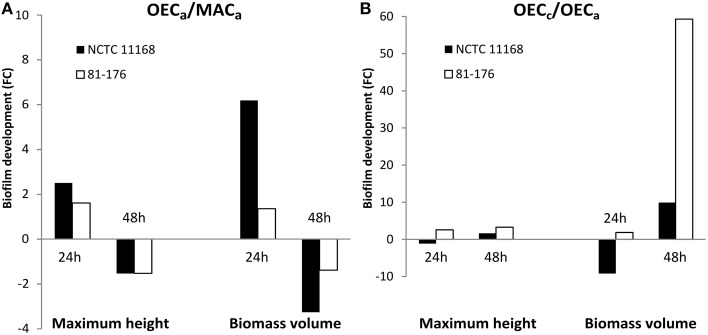
**Oxygen-enriched conditions enhanced biofilm development prior to and during biofilm formation of** ***C. jejuni***. Fold changes (FC) of biofilm development of *C. jejuni* strains NCTC 11168 (black bars) and 81-176 (white bars) as expressed by maximum height and biomass volume. **(A)** FC representing biofilm formation of cells acclimatized to OEC (OEC_*a*_: 19% O_2_, 10% CO_2_, 71% N_2_) and to MAC (MAC_a_: 5% O_2_, 10% CO_2_, 85% N_2_) prior to biofilm formation in MAC. **(B)** FC of biofilm development of cells submitted to OEC (OEC_*c*_) during biofilm cultivation and cells submitted to OEC (OEC_a_) prior to biofilm cultivation in MAC. Statistical data are presented in Supplementary Tables 2, 3.

In the second approach, both strains were acclimatized to OEC (OEC_a_) prior to biofilm formation in MAC. Acclimatized cells of both strains formed significantly larger biofilms than non-acclimatized ones after 24 h of cultivation, as expressed by the fold changes in maximum height and biomass volume values (Figure 3A). Conversely, the acclimatization of cells to OEC was no longer an advantage for biofilm formation after 48 h, as demonstrated by reduction of biofilm formation for both strains. This was also confirmed by statistical analyses, with the highest *F*-ratios of the interaction effect between “Incubation time” and “O_2_ pretreatment,” showing higher variation in maximum height and biomass volume, than for the other factors (Supplementary Table 2).

In order to distinguish the effect of OEC prior to or during *C. jejuni* biofilm formation, biofilm development was compared between the cells acclimatized to OEC and the cells subjected to OEC during biofilm formation (OEC_a_ and OEC_*c*_, respectively; Figure 3B). Although the fold change (OEC_*c*_/OEC_a_) was not in favor of NCTC 11168 biofilm formation during the first 24 h, after 48 h both strains cultivated in OEC_*c*_ showed enhanced biofilm formation with a marked difference in biomass volume for 81-176. This was confirmed statistically with a significant effect of OEC treatment (*P* < 0.0001) for O_2_ treatment, and the interaction between “Incubation time” and “O_2_ treatment” with the highest *F*-ratios (Supplementary Table 3).

### Role of *cosR* in biofilm development

**Figure 4:**
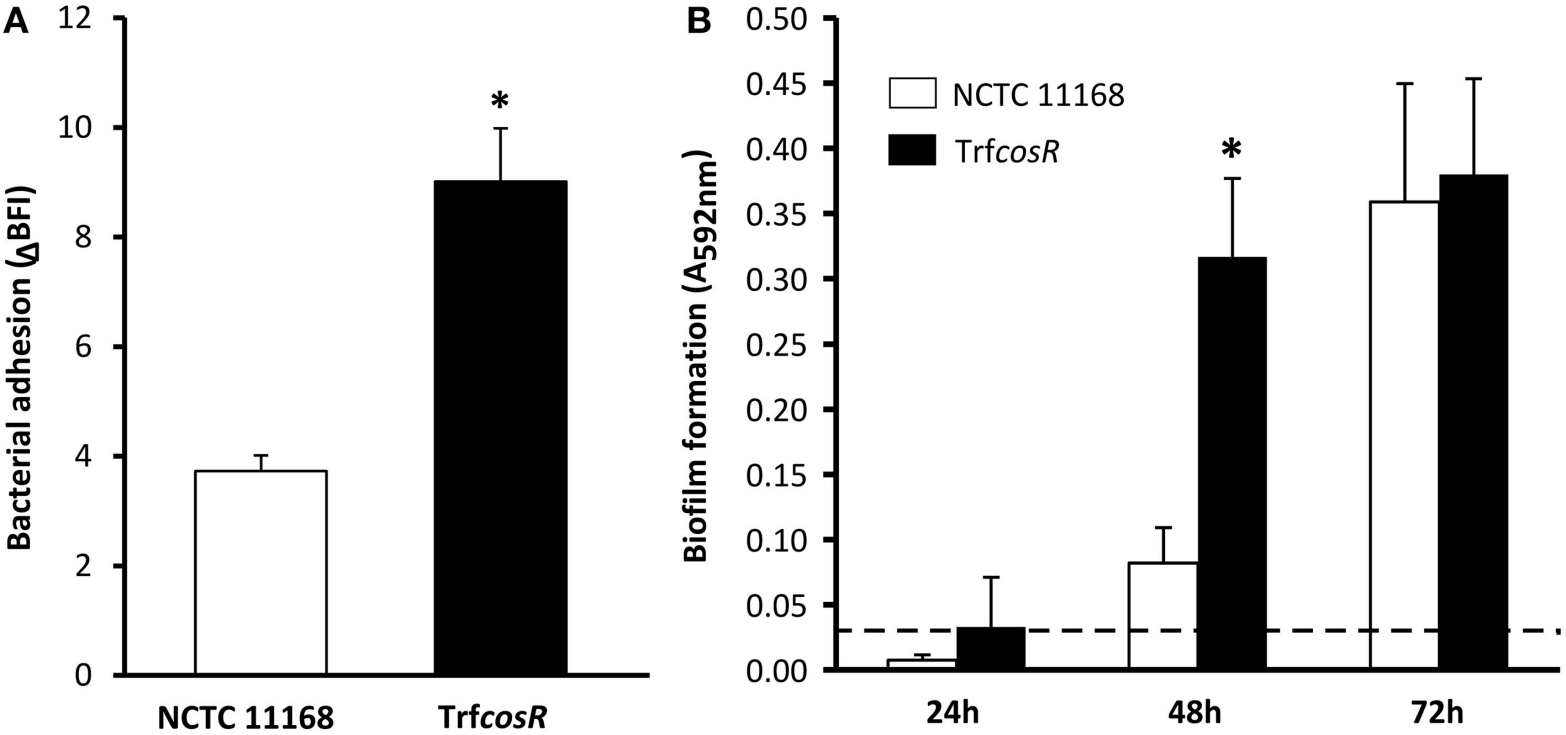
**The NCTC 11168** ***cosR*** **overexpressing transformant enhanced cell adhesion to inert surface and biofilm formation in comparison with its parental strain**. Adhesion to an inert surface **(A)** and biofilm formation **(B)** of *C. jejuni* NCTC 11168 (white bars) and the *cosR* overexpressing transformant (Trf*cosR*) (black bars) strains. Bacterial adhesion was determined after 2 h by calculating the BioFilm Index (BFI) using the BioFilm Ring Test®. Biofilm formation was measured in 24-well microtitre plates at 24, 48, and 72 h using the crystal violet assay. Error bars represent the standard deviation of three independent experiments. Asterisks indicate significant differences (*P* < 0.05) between the parental strain and the transformant. A dashed line represents detection limit of the crystal violet assay.

**Figure 5:**
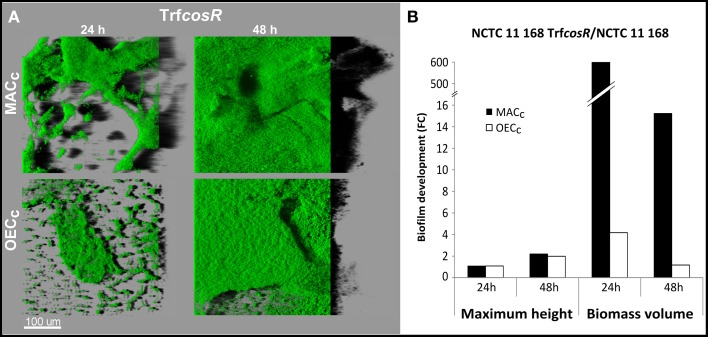
**The CosR is responsible for biofilm maturation in** ***C. jejuni***. Biofilm structure of *C. jejuni* NCTC 11168 and the *cosR* overexpressing transformant (Trf*cosR*) after incubation for 24 and 48 h in MAC (black bars) or OEC (white bars). **(A)** The CLSM images representing an aerial view of biofilm structures with the shadow projection on the right. **(B)** Trf*cosR* biofilm development in comparison to the parental strain expressed as a fold changes of maximum height and biomass volume. Statistical data are presented in Supplementary Tables 4, 5.

A second copy of *C. jejuni* gene *cosR* and its promoter were inserted into the poorer biofilm-forming strain NCTC 11168 to determine its role in *C. jejuni* biofilm formation. This construction, with an ectopic copy of the *cosR* gene and its promoter, enabled to double the expression of the transcript level of *cosR* in the cells (Supplementary Figure 1) in a same manner as in the *cosR*-overexpressing strain obtained by Hwang et al. (2011) and used by Oh and Jeon (2014). Then, the parental NCTC 11168 strain and the *cosR* overexpressing strain, namely transformant (Trf*cosR*), were compared for their ability to adhere to an inert surface and to develop a biofilm (Figure 4). Using the BioFilm Control Ring Test®, a significantly higher ΔBFI was obtained (*P* = 0.0007) for the transformed strain, indicating its greater ability to adhere to inert surfaces (Figure 4A). In addition, using the crystal violet assay, the transformant showed enhanced biofilm formation after 24 and 48 h (*P* = 0.0006 and 0.02, respectively) but not after 72 h (*P* > 0.05) when compared with its parental strain (Figure 4B). The CLSM observations and biofilm analyses indicated that the transformant formed significantly more (*P* < 0.01) biofilm than its parental strain (Figure 5, Supplementary Table 4). In addition, the maximum height and biomass volume reached by the transformant was not significantly different from those obtained with the strongest biofilm-forming strain 81-176 (Supplementary Table 5). These data showed that the presence of two genes encoding *cosR* significantly enhanced biofilm development in MAC (592.7-times higher biomass volume after 24 h). Interestingly, this was correlated with the formation of an open biofilm ultrastructure with voids and water channels similar to the one described for 81-176 (Figures 1, 5A). Comparison of genomic sequences using xBASE2 (Chaudhuri et al., 2008) showed that the *cosR* gene (*cj0335c* and *cjj0379c*, respectively) and its flanking regions are 100% identical in NCTC 11168 and 81-176. Both strains carry the exact same form of the gene. Therefore, some other mechanisms, related to the *cosR* sequence and its flanking regions, for regulating the *C. jejuni* biofilm formation, should exist. Moreover, unlike the two wild strains, an increased O_2_ concentration during cultivation did not promote biofilm formation of the transformant (Figure 5). These data indicate that a second ectopic copy of *cosR* enhanced biofilm development by promoting a complex architecture of *C. jejuni* biofilm irrespective of O_2_ demand. Nevertheless, further experiments should be performed to evaluate *cosR* transcript level and CosR expression throughout all phases of biofilm development.

## Discussion

As the leading cause of bacterial foodborne diseases, whose incidence has been significantly increasing during the recent years in Europe (EFSA, 2010, 2012, 2013), this pathogen has to adapt and survive environmental conditions outside and inside its main hosts, particularly oxidative stress. In this study, we have shown that *C. jejuni* can form biofilm in static conditions with a clearly defined finger-like structure. Our observation is consistent with previous studies indicating that *C. jejuni* could develop monospecies biofilms (Kalmokoff et al., 2006; Asakura et al., 2007; Corcoran and Moran, 2007; Reeser et al., 2007; Fields and Thompson, 2008; Hanning et al., 2008; McLennan et al., 2008; Sanders et al., 2008; Gunther and Chen, 2009). Both examined strains were able to produce a biofilm, although their maximum height, biomass volume, and ultrastructure differed significantly between the two strains. In previous studies, stronger adhesion to an inert surface was observed for 81-176 than for NCTC 11168 (Gunther and Chen, 2009; Sulaeman et al., 2010; Teh et al., 2010). Although the adhesion strength could not be fully correlated to the capability of bacterial species to form biofilms, biofilm initiation is crucial to anchor the embryonic core of the biofilm. Our qualitative and quantitative data indicated that NCTC 11168 formed a thin but compact multilayered biofilm without achieving a more complex organization during the time of incubation. In contrast, the 81-176 strain was able to form a thick biofilm with an open ultrastructure composed of voids and channels. This kind of heterogeneous structure is considered to be the signature of a mature biofilm. It enhances the formation of convective flows bringing nutrients to cell aggregates and draining metabolic waste from cells in these aggregates (Donlan and Costerton, 2002). The heterogeneity of the 81-176 biofilm was confirmed by tracking the motile cells within the *C. jejuni* 81-176 biofilm. In contrast to many other bacteria, *C. jejuni* is able to maintain the expression level of genes responsible for cell motility and flagella biosynthesis when grown in biofilms (Joshua et al., 2006; Kalmokoff et al., 2006; Asakura et al., 2007; Reeser et al., 2007). In our study, we observed the presence of motile, less motile and sessile cells, indicating that the biofilm is composed of cells in different physiological states. Due to the biofilm organization, different cell phenotypes coexist in the structure and therefore a wide range of cells can be found in the biofilm, from dormant to motile cells. As in nature (*ex vivo* or *in vivo*) *C. jejuni* cells may encompass various physiological states, biofilm could be considered as a model of mixed subpopulations of *C. jejuni* which could be found in food products, food-processing plants, in poultry gut, or human digestive tract.

Although *C. jejuni* is sensitive to increased concentrations of oxygen, absence of oxygen in anaerobic conditions induces cell death. *C. jejuni* requires a basal amount of available oxygen to maintain the processes essential for respiration and multiplication (Kelly, 2008). The availability of dissolved oxygen is therefore one of the main environmental parameters for the survival of *C. jejuni*. Previous studies showed that aerobic conditions enhanced biofilm formation of the strain NCTC 11168 (Asakura et al., 2007; Reuter et al., 2010). In those studies, biofilms grown in glass tubes or in 24 well plates were detected by crystal violet or Congo red after exposition to air and air supplemented with CO_2_. These colorimetric assays showed enhancement of biofilm formation under the oxidative stress, but could not predict whether and how the biofilm structure would change. The controlled O_2_ gaseous conditions, respecting the capnophilic nature of *C. jejuni*, and the use of CLSM allowed us not only to quantify the amount of biofilm, but also to evaluate any structural changes caused by increased oxygen concentration. In accordance to our expectations, we did observe an increased biofilm formation for both strains under OEC. Moreover, the data obtained using CLSM suggest that the response to an increased O_2_ level is strain-dependent. Although the biofilm formation for both strains was enhanced, the ultrastructures were remarkably different. The poorer biofilm forming NCTC 11168 produced more voluminous biofilm without increasing its thickness and without switching to a maturation phase as observed for 81-176. As the physiological state of cells may correspond to their close environment, the cell response to environmental conditions could differ according to its location in the biofilm structure. The formation of voluminous flat biofilm may be beneficial for NCTC 11168 under OEC, as a smaller area, and therefore a smaller number of cells, is exposed to the malignant effect of oxygen. On the other hand, the 81-176 strain increased in both biofilm volume and height, keeping the porous ultrastructure of the biofilms produced under MAC. It seems like the strain disregards the negative effects of an increased oxygen level and is supported to multiply and form a mature biofilm composed of mixed subpopulations of cells. The biofilm organization may therefore offer a favorable oxygen tuning niche for *C. jejuni*. These findings indicate that oxygen growth requirements of *C. jejuni* are not as fastidious when cells are organized in biofilm. Consequently, the paradigm of fastidious requirements for *C. jejuni* growth (Jones, 2001; Park, 2002) should be reconsidered according to the cell physiological state and cell population cooperation.

Unlike well-studied aerobes, *C. jejuni* lacks specific and global regulators involved in oxidative stress resistance, such as SoxRS, OxyR, or RpoS (Garenaux et al., 2008). *C. jejuni* carries two Fur homologs, Fur, and PerR (peroxide stress regulator), which regulate iron homeostasis and contribute to the oxidative stress response (Van Vliet et al., 2002). Recently, Hwang et al. (2011, 2012) have suggested that the orphan TCS Cj0355c could be involved in the oxidative stress response and named it CosR. The protein product of *cosR* shares 60% amino acid identity with Hp1043, a TCS response regulator element from the close relative *Helicobacter pylori*. Deletion of *hp1043* induced death of *H. pylori* in the same way as it has been observed for *cosR* and *C. jejuni* (Stahl and Stintzi, 2011). However, the *hp1043* gene has been successfully substituted by *C. jejuni cosR* (Muller et al., 2007) suggesting that CosR exhibits similar biological functions to Hp1043.

In this study, the essential gene *cosR* was overexpressed in the poorer biofilm-forming strain, NCTC 11168, in order to investigate the role of this TCS in biofilm formation and structuring. In our study, the adhesion of cells to inert surfaces was correlated to the biofilm formation detected by Crystal violet and analyzed by CLSM. All three different detection techniques led to the same conclusion. The significantly greater adhesion to an inert surface and the increased biofilm formation of the transformant (Trf*cosR*) revealed that the expression of this gene is connected to biofilm formation. This was also confirmed by the CLSM experiments, which showed a much greater thickness and volume of the transformant's biofilm under MAC than those observed for the parental strain. This result is in accordance with the previously published work describing increased expression of CosR in *C. jejuni* NCTC 11168 biofilm as compared to planktonic counterparts (Kalmokoff et al., 2006), although Oh and Jeon (2014) observed decrease of biofilm formation in strain overexpressing *cosR*. This discrepancy might be explained by looking at the structure of biofilms of parental strain and the transformant. Interestingly, the ultrastructure of the Trf*cosR* biofilm was found to be more similar to the one described for 81-176 than the parental strain, showing an open organization with pores and channels across the structure. Unexpectedly, when the transformant was cultivated under OEC, the maximum height and biomass volume of the biofilm were not higher than when the biofilm was produced under MAC. Nevertheless, the values still remained higher than those of the parental strain. These data indicate that cells overexpressing *cosR* were not stimulated by the higher O_2_ concentrations to enhance the biofilm formation. Thus, CosR seems to be crucial for initiation of the maturation phase of *C. jejuni* biofilm development. This might be the reason of arisen discrepancy between our work and the one published by Oh and Jeon (2014). The authors used Mueller Hinton broth and higher temperature of cultivation. These factors were previously found to increase the biofilm formation of *C. jejuni* (Reeser et al., 2007). The usage of supportive cultivation conditions in combination with enhanced initiation of biofilm maturation caused by overexpression of CosR might result in earlier dispersal of cells and microcolonies from mature biofilm. Such acceleration of dispersal would result in reduction of biofilm mass attached to the surface of the well and *cosR* transformant would therefore seem to be less biofilm forming. This is in accordance with our observation of the dramatic biovolume decrease after 48 h of cultivation (from 600- to 16-times more biofilm mass than the parental strain) observed for the transformant. Nevertheless, further experiments should be performed in order to confirm or refuse this hypothesis.

The regulator CosR was initially identified as a potential regulator of ROS scavengers by promoting or repressing genes encoding KatA, AhpC, and SodB in *C. jejuni* (Hwang et al., 2011, 2012). It was also differently expressed after a superoxide stress induced by paraquat (Garenaux et al., 2008). Binding to the promoter of *luxS*, CosR might also be contributing to a quorum sensing system (Hwang et al., 2011). Recently, it was also demonstrated that CosR is involved in the expression of the antibiotic efflux pump CmeABC in *C. jejuni* (Hwang et al., 2012). In this study, role of CosR in the maturation of *C. jejuni* biofilm, independently of oxidative stress, adds a new element in favor of its pleiotropic function in the main metabolic processes allowing the survival of *C. jejuni* in response to environmental stresses.

In contrast to its highly restricted catabolic capacity, *C. jejuni* is able to develop strategies to survive environmental oxidative stress using O_2_ as an advantage for biofilm development. As *C. jejuni* is equipped to withstand oxidative stress through cooperation of subpopulations within a biofilm, further analyses are required to assess if this feature could explain the survival of this emerging pathogen in slaughterhouses, after evisceration, during food processing, or during macrophage attack. In addition, these findings advocate further studies to analyze quiescent, dormant and sessile *C. jejuni* cells and cell cooperation in response to environmental stresses, to identify the underlying cellular and molecular mechanisms supporting the persistence and resistance of this mysterious pathogen.

## Materials and methods

### Bacterial strains and culture conditions

All experiments were performed using three strains of *C. jejuni*: two well-documented clinical isolates 81-176 and NCTC 11168 purchased from general collections, and a *cosR* overexpressing transformant built for this study as described below. All strains were subcultured from the stock stored at −80°C by cultivation on Karmali agar plates (Oxoid, UK) at 42°C for 48 h in MAC (5% O_2_, 10% CO_2_, and 85% N_2_, namely MAC). Grown colonies were inoculated onto Karmali agar plates and incubated either for 24 h at 42°C in MAC, or for 42 h in oxygen-enriched conditions (19% O_2_, 10% CO_2_, and 71% N_2_, namely OEC) to allow acclimation of cells to oxidative stress. The OEC were previously described as a sublethal atmosphere not repressing growth of *C. jejuni* NCTC 11168 and 81-176 (Sulaeman et al., 2012). The gas conditions were maintained using hermetic stainless steel jars vacuum flushed and then filled with commercially purchased gas mixture. The process was repeated two times to minimize air residua in the cultivation atmospheres. The growth rates of all tested strains were determined from cultivation in BHI (Merck, Germany) in MAC using plate counts in triplicates with the appropriated decimal dilution.

### Construction of the *cosR* overexpressing strain

For the construction of the *cosR* overexpressing strain, the cj0355c (*cosR*) gene was amplified from the strain NCTC 11168 using PCR primers Cj0355c F and Cj0355c R (Supplementary Table 6). The positions of the forward and reverse primers were chosen upstream and downstream of *cosR* within the *folB* (start position at 325186) and *fdxB* (end position at 323902) genes, respectively, to ensure that *cosR* was under the control of its own promoter. The PCR product was purified using the Qiagen PCR purification kit (Toronto, ON, Canada) and then cloned into the pRRK-1 plasmid (Reid et al., 2008). The cloning step was achieved using the Clontech In-Fusion™ PCR cloning kit (Mountain View, CA, USA). Briefly, the primers were designed with 15 bp extensions that allow recombination with the nucleotides flanking the XbaI restriction site on the pRRK vector. The recombinant pRRK + *cosR* plasmid was transformed into Fusion-Blue competent cells and positive transformants were selected on LB agar plates supplemented with Km. The cloned plasmid with the *cosR* gene was extracted from the grown transformants, purified, and sequenced to confirm the absence of point mutations. The plasmid was then naturally transformed into *C. jejuni* NCTC 11168 grown to mid-log phase. Following incorporation of the *cosR* into the chromosome was achieved by heterologous recombination. The location of the inserted gene was determined by amplifying three possible insertion sites on the chromosome using the ak233, ak234, ak235, and AR56 primers (Supplementary Table 6). The expected PCR product size was detected using the ak234 and AR56 primers indicating that *cosR* was inserted downstream of *cj0431*. The NCTC 11168 + *cosR* + Km^R^ strain is henceforth referred to as the *cosR* overexpression transformant or, for simplicity, the “transformant” or “Trf*cosR*.” The growth rates of the parental NCTC11168 strain and the transformant were similar (μ_max_ = 0.69 and 0.72 h^−1^, respectively). The overexpression of *cosR* was validated using quantitative RT-PCR after RNA extraction according to Sulaeman et al. (2012) with the following modifications. The quantity of total RNA was assessed using a Nanodrop 2000 (Thermo Fisher Scientific, Courtaboeuf, France), and the integrity of the RNA was verified with an Experion™ Automated Electrophoresis Station (BioRad) using the Experion RNA StdSens Analysis Kit (BioRad) according to the manufacturer's guidelines. Absence of DNA in the samples was confirmed by PCR with primers targeting *flaA* (Supplementary Table 6). Only high quality RNA samples without DNA contamination were used in qRT-PCR assays.

### Adhesion to an inert surface

The adhesion capability of *C. jejuni* strains was determined using the BioFilm Ring Test® (BioFilm Control, France) as described previously by Sulaeman et al. (2010). Briefly, each culture was pelleted and resuspended in buffered peptone water, the OD_600nm_ was adjusted to 1 and suspension was used for inoculation of plate wells. After 2 h of cultivation under MAC at 42°C, adhesion was determined by measuring BFI (Biofilm Index) using the BioFilm Control developed software. The BFI correlates to the number of magnetic microbeads detected after well magnetization. The ΔBFI was calculated by subtracting the BFI of blank control from the BFI of the sample. The assay was repeated three times with three technical replicates for each independent culture.

### Biofilm formation

The crystal violet biofilm assay was used to determine the amount of biofilm produced by *C. jejuni*. The protocol was adapted from that described by Djordjevic et al. (2002). Briefly, 2 ml of *C. jejuni* suspension was inoculated in 24-well sterile microtitre plates. Each plate was incubated statically for 24, 48, or 72 h at 42°C in MAC. After cultivation, planktonic cells were washed out and biofilm was stained with 1% crystal violet solution. The crystal violet bound to the biofilm was then eluted using 99% ethanol and the absorbance of the eluate was measured at 595 nm.

Qualitative (ultrastructural) and quantitative data (maximum thickness and biomass volume) of *C. jejuni* biofilm were measured on biofilm produced in 96-well polystyrene microtitre plates with a μ clear® base (thickness of 190 ± 5 μm; Greiner Bio-one, Germany). Prior to the inoculation of microtitre plates, grown cells were transferred from Karmali plates into BHI, washed once and resuspended in sterile BHI to final OD_600nm_ = 0.8 ± 0.05. The suspension was then loaded onto the microtitre plate in triplicates for each strain (250 μl per well). The plates were incubated in MAC or OEC for 4–5 h at 37°C allowing *C. jejuni* cells to adhere to the substratum. After that, the bacterial suspension was carefully replaced with 250 μl of sterile BHI and microtitre plates were incubated for the next 24 and 48 h at 37°C in MAC or OEC, depending on the experiment. The μ clear® base material allows diffusion of gas molecules into the liquid media and therefore ensures formation of biofilm that is attached to the bottom of the well and not floating on the air-liquid interphase. Finally, wells containing biofilm were stained using 50 μl of Syto 9 solution (Invitrogen, USA) diluted in BHI to the final concentration of 2 μl/ml. The Syto 9 is cell-permeable dye intercalating with DNA and therefore staining the cells and the eDNA of biofilm matrix. All biofilms were observed using confocal laser scanning microscope (CLSM) as described below. For each condition, three independent replicates were analyzed.

### Confocal laser scanning microscopy (CLSM) image acquisition

Each well of the microtitre plates was scanned using the inverted Leica SP2 AOBS confocal laser scanning microscope (LEICA Microsystems, Germany) at 400 Hz with a 40x/0.8 water immersion objective lens Leica HCX Apo. The fluorophore Syto 9 was excited with a 488-nm argon laser. The whole well area was inspected to verify the presence of biofilm, then the most representative place was scanned providing a stack of horizontal planar images (512 × 512 pixels representing an area of 375 × 375 μm) with a z-step of 1 μm. At least one stack of horizontal planar images was acquired for each replicate.

### Image analysis

The stacks obtained from the microscopic observations were processed using Imaris 7.6.4 software (Bitplane, Switzerland). Images representing an aerial view of biofilm structure were rendered using the Easy 3D view with the auto-adjustment function to correct pixel intensities. Numerical data and 3D models of the biofilm structures were generated using the surface generator function of the Measurement Pro module with the minimal threshold set at 40 for the green channel (Syto 9). Only objects bigger than 10 voxels were included in the analysis. Biofilm development was normalized according to height (thickness determined from z-stacks as the last image showing consecutive signal from biofilm structures) and biomass volume (cell abundance).

### Statistical analyses

The numerical data obtained from Imaris were processed with STATGRAPHICS Centurion 16.1.11 software (StatPoint, Inc., Herndon, VA, USA) with the maximum height (biofilm thickness) and the biomass volume (cell abundance) as explanatory values. For all variance analyses, ANOVAs were performed to determine the individual effect of each factor and potential interacting effects with the confirmation of a normal distribution for each data set.

Assay variations were excluded from interacting effects, as they were not significantly different at the first order. The significance level was selected at 99%, consequently an effect was considered significant if its *P*-value was lower than 0.01. All *F*-*ratios* were based on the average residual squared error. When the transformant (Trf*cosR*) was used, a multiple comparison using the Scheffé method was implemented in ANOVAs to classify the significant variations (at 95% confidence) according to the strains.

For cell adhesion to an inert surface and crystal violet biofilm assays, significant differences were determined using two-sided Student's *t*-test comparisons at a 95% significance level with the confirmation of a normal distribution for each data set.

## Author contributions

HT, RB, and OT conceived and designed the study. NH, MH, and AS built and validated the transformant. HT, RR, and RB performed the experimental work. HT prepared the manuscript and RB and OT contributed to the final manuscript.

### Conflict of interest statement

The authors declare that the research was conducted in the absence of any commercial or financial relationships that could be construed as a potential conflict of interest.

## Abbreviations

BFI: Biofilm index
CLSM: Confocal laser scanning microscopy
MAC: Microaerobic conditions
OEC: Oxygen-enriched conditions
OEC_*C*_: Cultivation in OEC
OEC_A_: Acclimation to OEC
ROS: Reactive oxygen species
TCS: Two-component system.
