# Supplementary material for: Biofilm spatial organization by the emerging pathogen Campylobacter jejuni: comparison between NCTC 11168 and 81-176 strains under microaerobic and oxygen-enriched conditions
Source: Front Microbiol. 2015 Jul 13;6:709. doi: 10.3389/fmicb.2015.00709 (PMC4499754; doi:10.3389/fmicb.2015.00709)
Supplement: Supplementary file 1 [file Table1.DOCX]

| **S1 Table.** **Significance of factors and their interactions as a function of O_2_ treatment during biofilm formation.** Factors calculated by ANOVA of *C. jejuni* biofilm formation according to biofilm thickness (maximum height) and the cell abundance (biomass volume). Analyzed factors: Strains (NCTC 11168/81-176), assays (1/2/3), incubation time (24 h/48 h) and O_2_ concentration during biofilm formation (MAC_c_/OEC_c_). | | | | | | | | | | | | | | | | | | | | |  |
| --- | --- | --- | --- | --- | --- | --- | --- | --- | --- | --- | --- | --- | --- | --- | --- | --- | --- | --- | --- | --- | --- |
| **Maximum height** | | | | | | | | | | | | | | | | | | | | | |
| Source | Sum of Squares | | | | | Df | | | | Mean Square | | | *F*-ratio | | | | *P*-value | | | | |
| *Main effects* | |  | |  | | | | |  | | |  | | | |  | | | |  |  |
| Strain | | 88695.00 | | 1 | | | | | 88695.00 | | | 22.15 | | | | 0.0003 | | | |  |  |
| Assay | | 8412.75 | | 2 | | | | | 4206.38 | | | 1.05 | | | | 0.3741 | | | |  |  |
| Incubation time | | 19323.40 | | 1 | | | | | 19323.40 | | | 4.83 | | | | 0.0442 | | | |  |  |
| O_2_ concentration | | 5251.04 | | 1 | | | | | 5251.04 | | | 1.31 | | | | 0.2700 | | | |  |  |
| *Interactions* | |  | |  | | | | |  | | |  | | | |  | | | |  |  |
| Strain x Incubation time | | 9087.04 | | 1 | | | | | 9087.04 | | | 2.27 | | | | 0.1527 | | | |  |  |
| Strain x O_2_ concentration | | 12015.40 | | 1 | | | | | 12015.4 | | | 3.00 | | | | 0.1037 | | | |  |  |
| Incubation time x O_2_ concentration | | 715.04 | | 1 | | | | | 715.042 | | | 0.18 | | | | 0.6786 | | | |  |  |
| Residual | | 203553.00 | | 15 | | | | | 4003.53 | | |  | | | |  | | | |  |  |
| Total (corrected) | | 6.67 x 10^13^ | | 23 | | | | |  | | |  | | | |  | | | |  |  |
| **Biomass volume** | | | | | | | | | | | | | | | | | | | | | |
| Source | | | Sum of Squares | | | | Df | | | | Mean  Square | | | | *F*-ratio | | | *P*-value | | |  |
| *Main effects* | | |  | |  | | |  | | | | | |  | | | | |  | |  |
| Strain | | | 13.30 x 10^12^ | | 1 | | | 13.30 x 10^12^ | | | | | | 13.06 | | | | | 0.0026 | |  |
| Assay | | | 0.52 x 10^12^ | | 2 | | | 0.26 x 10^12^ | | | | | | 0.25 | | | | | 0.7802 | |  |
| Incubation time | | | 30.31 x 10^12^ | | 1 | | | 30.31 x 10^12^ | | | | | | 29.62 | | | | | 0.0001 | |  |
| O_2_ concentration | | | 12.23 x 10^12^ | | 1 | | | 12.23 x 10^12^ | | | | | | 11.94 | | | | | 0.0035 | |  |
| *Interactions* | | |  | |  | | |  | | | | | |  | | | | |  | |  |
| Strain x Incubation time | | | 2.97 x 10^12^ | | 1 | | | 2.97 x 10^12^ | | | | | | 2.91 | | | | | 0.1087 | |  |
| Strain x O_2_ concentration | | | 47.83 x 10^12^ | | 1 | | | 0.48 x 10^12^ | | | | | | 0.47 | | | | | 0.5044 | |  |
| Incubation time x O_2_ concentration | | | 3.11 x 10^12^ | | 1 | | | 3.11 x 10^12^ | | | | | | 3.05 | | | | | 0.1013 | |  |
| Residual | | | 15.30 x 10^12^ | | 15 | | | 1.02 x 10^12^ | | | | | |  | | | | |  | |  |
| Total (corrected) | | | 78.28 x 10^12^ | | 23 | | |  | | | | | |  | | | | |  | |  |
