# Supplementary material for: Biofilm spatial organization by the emerging pathogen Campylobacter jejuni: comparison between NCTC 11168 and 81-176 strains under microaerobic and oxygen-enriched conditions
Source: Front Microbiol. 2015 Jul 13;6:709. doi: 10.3389/fmicb.2015.00709 (PMC4499754; doi:10.3389/fmicb.2015.00709)
Supplement: Supplementary file 2 [file Table2.DOCX]

**S2 Table.** **Significance of factors and their interactions as a function of O_2_ cell pretreatment prior to biofilm formation.** Factors calculated by ANOVA of *C. jejuni* biofilm formation according to biofilm thickness (maximum height) and the cell abundance (biomass volume). Analyzed factors: strains (NCTC 11168/81-176), assays (1/2/3), incubation time (24 h/48 h) and O_2_ pretreatment prior to biofilm formation in MAC (OEC_a_/MAC_c_).

| **Maximum height** |  |  |  |  |  |
| --- | --- | --- | --- | --- | --- |
| Source | Sum of Squares | Df | Mean Square | *F*-ratio | *P*-value |
| *Main effects* |  |  |  |  |  |
| Strain | 1434.38 | 1 | 1434.38 | 3.66 | 0.0749 |
| Assay | 583.36 | 2 | 291.68 | 0.74 | 0.4916 |
| Incubation time | 7088.47 | 1 | 7088.47 | 18.10 | 0.0007 |
| O_2_ concentration | 11.29 | 1 | 11.29 | 0.03 | 0.8675 |
| *Interactions* |  |  |  |  |  |
| Strain x Incubation time | 4524.86 | 1 | 4524.86 | 11.55 | 0.0040 |
| Strain x O_2_ pretreatment | 1210.69 | 1 | 1210.69 | 3.09 | 0.0991 |
| Incubation time x O_2_ pretreatment | 14188.30 | 1 | 14188.30 | 36.23 | <0.0001 |
| Residual | 5874.59 | 15 | 391.64 |  |  |
| Total (corrected) | 34915.90 | 23 |  |  |  |
| **Biomass volume** |  |  |  |  |  |
| Source | Sum of Squares | Df | Mean Square | *F*-ratio | *P*-value |
| *Main effects* |  |  |  |  |  |
| Strain | 2.86 x 10^11^ | 1 | 2.86 x 10^11^ | 6.79 | 0.0199 |
| Assay | 0.60 x 10^11^ | 2 | 0.60 x 10^11^ | 0.71 | 0.5079 |
| Incubation time | 2.67 x 10^11^ | 1 | 2.67 x 10^11^ | 6.34 | 0.0236 |
| O_2_ pretreatment | 1.70 x 10^11^ | 1 | 1.70 x 10^11^ | 4.04 | 0.0628 |
| *Interactions* |  |  |  |  |  |
| Strain x Incubation time | 3.45 x 10^11^ | 1 | 3.45 x 10^11^ | 8.21 | 0.0118 |
| Strain x O_2_ pretreatment | 0.34 x 10^11^ | 1 | 0.34 x 10^11^ | 0.80 | 0.3845 |
| Incubation time x O_2_ pretreatment | 13.05 x 10^11^ | 1 | 13.05 x 10^11^ | 30.99 | <0.0001 |
| Residual | 6.31 x 10^11^ | 15 | 6.31 x 10^11^ |  |  |
| Total (corrected) | 31.08 x 10^11^ | 23 |  |  |  |
