# Supplementary material for: Biofilm spatial organization by the emerging pathogen Campylobacter jejuni: comparison between NCTC 11168 and 81-176 strains under microaerobic and oxygen-enriched conditions
Source: Front Microbiol. 2015 Jul 13;6:709. doi: 10.3389/fmicb.2015.00709 (PMC4499754; doi:10.3389/fmicb.2015.00709)
Supplement: Supplementary file 3 [file Table3.DOCX]

**S3 Table. Significance of factors and their interactions as a function of O_2_ treatment prior to or during biofilm formation.** Factors calculated by ANOVA of *C. jejuni* biofilm formation according to biofilm thickness (maximum height) and the cell abundance (biomass volume). Analyzed factors: Strains (NCTC 11168/81-176), assays (1/2/3), incubation time (24 h/48 h) and O_2_ treatment (OEC_a_/OECc).

| **Maximum height** |  |  |  |  |  |
| --- | --- | --- | --- | --- | --- |
| Source | Sum of Squares | Df | Mean Square | *F*-ratio | *P*-value |
| *Main effects* |  |  |  |  |  |
| Strain | 42129.60 | 1 | 42129.60 | 13.25 | 0.0024 |
| Assay | 1050.51 | 2 | 525.25 | 0.17 | 0.8492 |
| Incubation time | 4278.94 | 1 | 4278.94 | 1.35 | 0.2641 |
| O_2_ treatment | 56797.90 | 1 | 56797.90 | 17.86 | 0.0007 |
| *Interactions* |  |  |  |  |  |
| Strain x Incubation time | 10480.90 | 1 | 10480.90 | 3.30 | 0.0895 |
| Strain x O_2_ treatment | 40875.50 | 1 | 40875.50 | 12.86 | 0.0027 |
| Incubation time x O_2_ treatment | 10067.10 | 1 | 10067.10 | 3.17 | 0.0954 |
| Residual | 47690.70 | 15 | 3179.38 |  |  |
| Total (corrected) | 213371.00 | 23 |  |  |  |
| **Biomass volume** |  |  |  |  |  |
| Source | Sum of Squares | Df | Mean Square | *F*-ratio | *P*-value |
| *Main effects* |  |  |  |  |  |
| Strain | 3.90 x 10^12^ | 1 | 3.90 x 10^12^ | 9.53 | 0.0075 |
| Assay | 1.06 x 10^12^ | 2 | 0.53 x 10^12^ | 1.30 | 0.3024 |
| Incubation time | 7.74 x 10^12^ | 1 | 7.74 x 10^12^ | 18.89 | 0.0006 |
| O_2_ treatment | 18.85 x 10^12^ | 1 | 18.85 x 10^12^ | 46.00 | <0.0001 |
| *Interactions* |  |  |  |  |  |
| Strain x Incubation time | 0.19 x 10^12^ | 1 | 0.19 x 10^12^ | 0.46 | 0.5100 |
| Strain x O_2_ treatment | 5.41 x 10^12^ | 1 | 5.41 x 10^12^ | 13.21 | 0.0024 |
| Incubation time x O_2_ treatment | 19.73 x 10^12^ | 1 | 19.73 x 10^12^ | 48.14 | <0.0001 |
| Residual | 6.15 x 10^12^ | 15 | 0.41 x 10^12^ |  |  |
| Total (corrected) | 63.03 x 10^13^ | 23 |  |  |  |
