# Supplementary material for: Biofilm spatial organization by the emerging pathogen Campylobacter jejuni: comparison between NCTC 11168 and 81-176 strains under microaerobic and oxygen-enriched conditions
Source: Front Microbiol. 2015 Jul 13;6:709. doi: 10.3389/fmicb.2015.00709 (PMC4499754; doi:10.3389/fmicb.2015.00709)
Supplement: Supplementary file 4 [file Table4.DOCX]

**S4 Table. Significance of factors and their interactions as a function of O_2_ treatment during biofilm formation.** Factors calculated by ANOVA of *C. jejuni* biofilm formation according to biofilm thickness (maximum height) and the cell abundance (biomass volume). Analyzed factors: Strains (NCTC 11168/NCTC 11168 Trfc*osR*), assays (1/2/3), incubation time (24 h/48 h) and O_2_ treatment (MAC/OEC_c_).

| **Maximum height** |  | |  |  | | |  | |  |  |
| --- | --- | --- | --- | --- | --- | --- | --- | --- | --- | --- |
| Source | Sum of Squares | | Df | Mean Square | | | *F*-ratio | | *P*-value |  |
| *Main effects* |  | |  |  | | |  | |  |  |
| Strain | 29962.70 | | 1 | 29962.7 | | | 30.10 | | 0.0001 |  |
| Assay | 7109.08 | | 2 | 3554.54 | | | 3.57 | | 0.0539 |  |
| Incubation time | 24576.00 | | 1 | 24576.0 | | | 24.69 | | 0.0002 |  |
| O_2_ treatment | 580.16 | | 1 | 580.167 | | | 0.58 | | 0.4571 |  |
| *Interactions* |  | |  |  | | |  | |  |  |
| Strain x Incubation time | 37604.20 | | 1 | 37604.2 | | | 37.77 | | <0.0001 |  |
| Strain x O_2_ treatment | 4592.67 | | 1 | 4592.67 | | | 4.61 | | 0.0485 |  |
| Incubation time x O_2_ treatment | 1014.00 | | 1 | 1014.0 | | | 1.02 | | 0.3289 |  |
| Residual | 14933.10 | 15 | | | 995.539 |  | |  | | |
| Total (corrected) | 120372.00 | 23 | | |  |  | |  | | |
| **Biomass volume** |  | |  |  | | |  | |  |  |
| Source | Sum of Squares | | Df | Mean Square | | | *F*-ratio | | *P*-value |  |
| *Main effects* |  | |  |  | | |  | |  |  |
| Strain | 28.56 x 10^12^ | | 1 | 28.56 x 10^12^ | | | 9.03 | | 0.0089 |  |
| Assay | 3.97 x 10^12^ | | 2 | 1.98 x 10^12^ | | | 0.63 | | 0.5478 |  |
| Incubation time | 57.73 x 10^12^ | | 1 | 57.73 x 10^12^ | | | 18.25 | | 0.0007 |  |
| O_2_ treatment | 1.10 x 10^12^ | | 1 | 1.10 x 10^12^ | | | 0.35 | | 0.5639 |  |
| *Interactions* |  | |  |  | | |  | |  |  |
| Strain x Incubation time | 19.54 x 10^12^ | | 1 | 19.54 x 10^12^ | | | 6.18 | | 0.0252 |  |
| Strain x O_2_ treatment | 19.80 x 10^12^ | | 1 | 19.80 x 10^13^ | | | 6.26 | | 0.0244 |  |
| Incubation time x O_2_ treatment | 1.27 x 10^12^ | | 1 | 1.27 x 10^12^ | | | 0.40 | | 0.5358 |  |
| Residual | 47.45 x 10^12^ | | 15 | 3.16 x 10^12^ | | |  | |  |  |
| Total (corrected) | 179.42 x 10^12^ | | 23 |  | | |  | |  |  |
