# Supplementary material for: Biofilm spatial organization by the emerging pathogen Campylobacter jejuni: comparison between NCTC 11168 and 81-176 strains under microaerobic and oxygen-enriched conditions
Source: Front Microbiol. 2015 Jul 13;6:709. doi: 10.3389/fmicb.2015.00709 (PMC4499754; doi:10.3389/fmicb.2015.00709)
Supplement: Supplementary file 5 [file Table5.DOCX]

**S5 Table. Multiple comparison of biofilm thickness (maximum height) and cell abundance (biomass volume).** Results obtained by using Scheffé analysis at 95% for strain classification from ANOVA. Analyzed factors: Strains (NCTC 11168/NCTC 11168 Trf*cosR*/81‑176), assays (1/2/3), incubation time (24 h/48 h) and O_2_ treatment (MAC/OEC_c_).

| **Maximum height** | | | | | | | | | | | | | | | |  |  |  |
| --- | --- | --- | --- | --- | --- | --- | --- | --- | --- | --- | --- | --- | --- | --- | --- | --- | --- | --- |
| *Scheffé statistical analysis* | | | | | | | | | | | | | | | | |  |  |
| Strain | Counts | | | Mean  Square | | | SD✝ square | | | H group∞ | | | | |  |  |  |  |
| NCTC 11168 | 12 | | 113.08 | | | 17.65 | | | A | | | | |  |  |  |  |  |
| NCTC 11168 Trf*cosR* | 12 | | 183.75 | | | 17.65 | | | B | | | | |  |  |  |  |  |
| 81-176 | 12 | | 234.66 | | | 17.65 | | | B | | | | |  |  |  |  |  |
| *Contrast* | | Sig. | | | Difference | | | +/- Limits | | | | | | | | |  |  |
| 81-176 - NCTC 11168 | | * | | | 121.58 | | | 51.52 | | | | |  |  |  |  |  |  |
| 81-176 - Trf*cosR* | |  | | | 50.92 | | | 51.52 | | | | |  |  |  |  |  |  |
| NCTC 11168 - Trf*cosR* | | * | | | -70.67 | | | 51.52 | | | | | | | | |  |  |
| **Biomass volume** | | | | | | | | | | | | | | | |  |  |  |
| *Scheffé statistical analysis* | | | | | | | | | | | | | | | | |  |  |
| Strain | Counts | | | Mean  Square | | | SD✝  square | | | | | H group∞ | | | | | | |
| NCTC 11168 | 12 | | 0.71 x 10^6^ | | | 4.34 x 10^5^ | | | | | A | | | | | | |  |
| NCTC 11168 Trf*cosR* | 12 | | 2.68 x 10^6^ | | | 4.34 x 10^5^ | | | | | B | | | | | | |  |
| 81-176 | 12 | | 2.89 x 10^6^ | | | 4.34 x 10^5^ | | | | | B | | | | | | |  |
| *Contrast* | | Sig. | | | Difference | | | +/- Limits | | | | | | | | |  |  |
| 81-176 - NCTC 11168 | | * | | | 2.00 x 10^6^ | | | 1.60 x 10^6^ | | | | | | | | |  |  |
| 81-176 - Trf*cosR* | |  | | | -0.21 x 10^6^ | | | 1.60 x 10^6^ | | | | | | | | |  |  |
| NCTC 11168 - Trf*cosR* | | * | | | -2.18 x 10^6^ | | | 1.60 x 10^6^ | | | | | | | | |  |  |

* significant difference between strains for maximum heigth R-square = 76.7 % and for volume 69.2 %

✝ Standard Deviation, ∞ Homogeneous group
