# Supplementary material for: Biofilm spatial organization by the emerging pathogen Campylobacter jejuni: comparison between NCTC 11168 and 81-176 strains under microaerobic and oxygen-enriched conditions
Source: Front Microbiol. 2015 Jul 13;6:709. doi: 10.3389/fmicb.2015.00709 (PMC4499754; doi:10.3389/fmicb.2015.00709)
Supplement: Supplementary file 6 [file Table6.DOCX]

**S6 Table. Primers used in the study.**

| **Primer name** | **Sequence 5’-3’** | **Function** |
| --- | --- | --- |
|  |  |  |
| Cj0355c F | GATTTAGATGTCTAGTGGTATATTAGATTTCGAAAGAAGGAA | Construction and location of *cj0355c* construct |
| Cj0355c R | GGGGAAGCTTTCTAGCAGGCAGGCTCTGAAAAATC |  |
| ak233 | GCAAGAGTTTTGCTTATGTTAGCAG |  |
| ak234 | GAAATGGGCAGAGTGTATTCTCCG |  |
| ak235 | GTGCGGATAATGTTGTTTCTG |  |
| AR56 | CATCCTCTTCGTCTTGGTAGC |  |
|  |  |  |
| RpoAQ-Fw | CGAGCTTGCTTTGATGAGTG | Q-PCR |
| RpoAQ-Rev | AGTTCCCACAGGAAAACCTA |  |
| Cj0355F | TTTGAAAGCTGGAGCTGATG |  |
| Cj0355R | GGTTCCGCCAAGTCTTAGTC |  |
|  |  |  |
| CPYFLA_1 | GGATTTCGTATTAACACAAATGGTGC | Checking RNA purity |
| CPYFLA_2 | CTGTAGTAATCTTAAAACATTTTG |  |
